# Brain organoids and organoid intelligence from ethical, legal, and social points of view

**DOI:** 10.3389/frai.2023.1307613

**Published:** 2024-01-05

**Authors:** Thomas Hartung, Itzy E. Morales Pantoja, Lena Smirnova

**Affiliations:** ^1^Center for Alternatives to Animal Testing (CAAT), Health and Whiting School of Engineering, Johns Hopkins University, Baltimore, MD, United States; ^2^CAAT-Europe, University of Konstanz, Konstanz, Germany

**Keywords:** microphysiological systems, cognition, intelligence, social sciences, learning and memory, consciousness

## Abstract

Human brain organoids, aka cerebral organoids or earlier “mini-brains”, are 3D cellular models that recapitulate aspects of the developing human brain. They show tremendous promise for advancing our understanding of neurodevelopment and neurological disorders. However, the unprecedented ability to model human brain development and function *in vitro* also raises complex ethical, legal, and social challenges. Organoid Intelligence (OI) describes the ongoing movement to combine such organoids with Artificial Intelligence to establish basic forms of memory and learning. This article discusses key issues regarding the scientific status and prospects of brain organoids and OI, conceptualizations of consciousness and the mind–brain relationship, ethical and legal dimensions, including moral status, human–animal chimeras, informed consent, and governance matters, such as oversight and regulation. A balanced framework is needed to allow vital research while addressing public perceptions and ethical concerns. Interdisciplinary perspectives and proactive engagement among scientists, ethicists, policymakers, and the public can enable responsible translational pathways for organoid technology. A thoughtful, proactive governance framework might be needed to ensure ethically responsible progress in this promising field.

## Introduction

Human brain organoids derived from pluripotent stem cells have emerged as a groundbreaking model system to study neurodevelopment and model neurological diseases (Eiraku et al., 2008; Hogberg et al., 2013; Lancaster et al., 2013; Pamies et al., 2017; Pasca, 2018). These simplified 3D culture systems recapitulate features of the developing human brain, allowing unprecedented access to early stages of neural organization and functioning *in vitro* (Di Lullo and Kriegstein, 2017). Noteworthy, the earlier used term “mini-brain” has been debunked in more recent consensus terminology (Pașca et al., 2022). The organoid approach has tremendous potential for advancing basic research, drug screening, personalized medicine, and cell therapy for injuries or neurodegenerative disorders (Huang et al., 2017; Sloan et al., 2018). The future possibilities seem boundless, with organoids implanted in animal models to enhance functionality or integrated into brain–computer interfaces (Yang et al., 2020). However, the properties that make cerebral organoids scientifically powerful also raise unique ethical challenges not observed with organoids of other tissues that require careful consideration (Farahany et al., 2018).

This review discusses key issues regarding the current scientific status of brain organoids, conceptualizations of consciousness and mind–brain relationships, ethical and legal dimensions, governance matters, public perceptions, and responsible translational pathways. A nuanced, interdisciplinary framework is required to develop organoid technology responsibly while addressing concerns.

## The current state, promise, and potential of brain organoid research

Human pluripotent stem cell-derived brain organoids can self-organize into organized neural tissue, exhibiting discrete brain regions and cell diversity (Birey et al., 2017). Each organoid resembles the early developing human brain, although simpler in organization, cell composition, and size. Different protocols allow the generation of region-specific organoids, such as forebrain, midbrain, or hypothalamic organoids (Qian et al., 2017, 2018).

The current research focus is on modeling neurodevelopmental principles and neurological disease mechanisms using brain organoids (Di Lullo and Kriegstein, 2017; Mansour et al., 2018; Adlakha, 2023). Organoids allow human-specific aspects of early corticogenesis to be investigated, which could shed light on evolutionary expansion of the human neocortex (Otani et al., 2016). Limitations currently include immature cell types, lack of organized cortical layers, variability between organoids, and absence of vascularization (Di Lullo and Kriegstein, 2017; Trujillo et al., 2019).

Nonetheless, organoids can recapitulate features of certain developmental brain disorders, such as microcephaly in Zika infection (Cugola et al., 2016; Garcez et al., 2016). Ongoing advances are enhancing organoid maturity, functionality, and structural organization (Mariani et al., 2015; Pasca et al., 2015; Giandomenico and Lancaster, 2017; Qian et al., 2019). Future directions, such as incorporating other cell types, 3D patterning, and orthotopic transplantation, could allow further maturation and validation against human clinical knowledge, and animal models will be essential to demonstrate applicability (Sloan et al., 2017; Mansour et al., 2018).

Brain organoids derived from human pluripotent stem cells allow unprecedented modeling of human brain development, disorders, and functionality *in vitro*. This disruptive technology has observed rapid adoption across diverse fields, such as

- *Neurodevelopmental toxicity testing*: Organoids with human-specific ontogeny provide superior models over animals to assess effects of toxins on processes such as neural differentiation and network formation (Zhong et al., 2020; Modafferi et al., 2021).
- *Disease modeling*: Patient-specific organoids with disease mutations have replicated features of autism, Alzheimer’s, and other disorders to elucidate mechanisms (Koo et al., 2019; Eichmüller and Knoblich, 2022; Lu and Yang, 2022).
- *Infection studies*: Organoids permit modeling of human-specific neurotropic pathogens inaccessible in animals, including Zika, HIV, and SARS-CoV-2 (Barreras et al., 2023).
- *Personalized medicine*: Organoids can screen patient-tailored therapies, exemplified in glioblastoma drug testing (Plummer et al., 2019).
- *Mixture toxicity*: Controlled toxicant combinations in organoids are revealing combination effects relevant to human risk (own ongoing NIH- and FDA-funded research).
- *Organoid Intelligence*: Linking organoids with AI aims to generate brain-like cognition – an emerging concept paralleling early AI research (Smirnova et al., 2023a, see below).

Much progress has been made since early cerebral organoids from induced pluripotent stem cells were first described in 2013, with continuous refinements in culture methods (Pasca, 2018). Methods to enhance organoid maturity include extending culture periods, providing biochemical or biophysical cues, co-culturing with other cell types, and xenotransplantation into rodents to allow vascularization (Mansour et al., 2018). Efforts are underway to produce interconnecting “assembloids”. Integrating cerebral organoids with microfluidics and multi-electrode arrays enables drug testing, disease modeling, and interfacing with robotic systems (Yang et al., 2020). The future possibilities for engineered functionally enhanced brain organoids seem immense and rapid advances are bringing more sophisticated capabilities within reach. However, existing protocols still only partially recreate human cortical development. Limitations like fetal-state cells, variability, and lack of vascularization currently restrict organoid maturity. While cerebral organoids can recapitulate discrete brain regions, they lack whole-brain organization and connectivity. Vascularization, neuronal maturation, and cell-type diversity remain limited (Trujillo et al., 2019). Translating insights from cerebral organoid studies into clinical treatments will require overcoming these deficiencies. Ongoing advances are overcoming these through vascularization, directed patterning, bioreactors, and increased functional complexity. Future aspirations like hyper-physiological systems, bioprinting, sensors, and fusing with brain-computer-interfaces (BCIs) could transform applications.

In conclusion, brain organoids constitute a disruptive platform to model neurobiology in unprecedented human settings. Realizing their full potential requires harnessing convergent technologies from tissue engineering, microphysiological systems development, AI, and biosensing. With creative innovation, organoids could unlock a new era in neuroscience and biomedicine.

## The emerging field of organoid intelligence (OI)

Organic Intelligence (OI) refers to the development of biologically inspired intelligent systems using human brain organoids and other biological components. Thus, OI is a novel interdisciplinary field at the intersection of biological computing and brain-machine interface technologies, focusing on the development of computational models using 3D cultures of human brain cells, specifically brain organoids (Friston, 2023). This emerging field combines advances in stem cell biology, tissue engineering, biomaterials, microfluidics, electrophysiology sensors, and machine learning (Smirnova et al., 2023a,b). The overarching goal of OI is to leverage the computational capabilities of biological neural networks to develop new forms of intelligence and biocomputing. This concept leverages the unique processing capabilities of the human brain, which excels in handling complex and uncertain data, and surpasses machines in decision-making with large, heterogeneous datasets. OI is built on key technological advancements, including the groundbreaking induction of pluripotent stem cells (iPSC) from human somatic cells and the subsequent development of 3D brain organoids from these iPSCs.

These advancements in 3D organoid culture have led to more physiologically accurate and scalable models for brain functions. Additionally, microfluidic perfusion systems are being developed to substitute for vasculature in brain organoids, providing essential nutrients and removing waste, critical for maintaining organoid homeostasis and viability. Another vital component is the incorporation of 3D microelectrode arrays for recording electrophysiological outputs from brain organoids, enabling the exploration of their computational potential and learning mechanisms. Ethically, OI research necessitates an embedded ethics approach involving interdisciplinary collaboration to analyze the ethical aspects and ensure responsible development, to be discussed below. The data management challenges in OI are substantial, requiring efficient big data infrastructure and supercomputing capacity for the analysis of massive datasets generated by organoid-MEAs. Moreover, there is a need for the standardization of experimental data and metadata, robust processing pipelines, efficient data storage, and the development of multimodal datasets.

The OI approach is inspired by the remarkable information processing capabilities of the human brain, which remain unparalleled by even the most advanced conventional computers. This has motivated efforts to incorporate biological components into computing systems in order to emulate brain functions. Progress by combining neuronal cultures with AI brings research closer to establishing memory and learning in a dish. A study by Kagan et al. (2022) explores the learning ability of human neuronal cultures grown in a lab dish using a system called DishBrain. The study found that the neurons can learn and exhibit sentience when embodied in a simulated game-world (Smirnova and Hartung, 2022). The paper does not provide a detailed discussion on the ethical implications of the research, but it does emphasize the importance of adhering to ethical guidelines and regulations in conducting research involving *in vitro* neurons and hiPSCs.

The applications and potential of OI are vast, including the development of novel biocomputing models where brain organoids are interconnected with real-world sensors and output devices, trained using biofeedback, big data warehousing, and machine learning methods. OI systems could enable transformative advances in fields like neuroscience, medicine, robotics, and computing (Smirnova et al., 2023a). For example, organoid models that exhibit cognition could provide unprecedented insights into human brain disorders and new platforms for drug screening. Furthermore, OI could significantly contribute to our understanding of brain development and disorders, potentially aiding in identifying treatments for neurological conditions such as dementia. In computing, hybrid organic–inorganic systems may possess brain-like capabilities exceeding current AI, helping overcome limitations of conventional silicon hardware. This system could lead to enhanced decision-making, continuous learning during tasks, and improved energy and data efficiency. OI represents a pioneering step in the realm of biocomputing, combining the intricacies of biological and machine learning, with vast potential for computational neuroscience and ethical implications that necessitate careful consideration.

However, realizing this vision will require solving significant scientific and engineering challenges, as well as addressing complex ethical issues.

The key technological breakthroughs of stem cell biology and organoid engineering described above, combined with neural interfaces, (e.g., microelectrode arrays and meshes, shank electrodes etc.) and Machine Learning and Big Data have enabled the emergence of OI (Smirnova et al., 2023a,b): OI also builds on decades of research in neuroprosthetics and neuromorphic engineering seeking to interface the nervous system with computing devices. A fundamental goal now is to advance organoid engineering and neural tissue maturation to support robust long-term recordings and increasing network complexity. This could enable higher-level learning and memory functions analogous to those produced through synaptic plasticity mechanisms in the brain. Applications and potential benefits of OI span neuroscience, medicine, computing, robotics, and brain-machine interfaces (Smirnova et al., 2023a) and include:

- *Disease modeling*: Patient-derived organoids could replicate pathological features associated with neurological disorders and allow screening potential therapies.
- *Biocomputing*: Networks of biological neurons may be capable of specialized processing exceeding current AI for certain tasks. OI systems could complement silicon hardware.
- *Brain-computer integration*: Bidirectional brain-machine interfaces based on OI elements could enable new assistive technologies and enhanced human-computer symbiosis.
- *Robotics*: Organoid controllers could endow machines with more flexible real-world learning and decision-making abilities.
- *Regenerative medicine*: Maturing organoids combined with scaffolds and vascularization might enable tissue grafts to repair brain injuries.

Achieving such applications could drive progress in diagnosing and treating neurological diseases, understanding brain information processing, enhancing machine intelligence, and expanding human cognitive capabilities through neural augmentation.

Along with its disruptive potential, OI faces formidable obstacles, challenges and open questions (Hartung et al., 2023). Beside the many philosophical and ethical concerns that require ongoing discussions to be covered in this article, they include:

- How can organoid maturation, complexity and functionality be enhanced? Long-term maintenance, neuronal subtype diversity, active myelination, and vascular integration to allow scaling of the organoids remain challenges.
- Can organoids exhibit higher-order network dynamics supporting robust learning and memory? This likely requires replicating complex neural connectivity found in the brain through advances in tissue engineering.
- What machine learning and computational analytics approaches are required to decode and interface with biological neural networks? Brain activity produces vast, multimodal datasets.
- How will OI systems be trained and implement learning algorithms very different from conventional deep learning? New techniques may need to be invented leveraging biological learning mechanisms.
- Can intelligent organoid systems be made reliably and not prone to dysfunction or uncontrolled growth? Strict quality control and safety precautions are needed.

Continued multidisciplinary collaboration across fields ranging from bioengineering and neuroscience to ethics will be needed to address these multifaceted opportunities and challenges (Magliaro and Ahluwalia, 2023). The Baltimore Declaration toward OI calls for this (Hartung et al., 2023): The Baltimore Declaration calls upon the scientific community to explore the potential of using human brain organoid cell cultures to advance understanding of the brain and develop new forms of biological computing, while proactively addressing ethical concerns. It coins the term “organoid intelligence” (OI) to describe this approach, drawing an analogy to artificial intelligence (AI). OI could enable breakthrough applications in areas like elucidating human cognitive functions, novel computing paradigms exceeding limitations of silicon hardware, advanced brain-machine interfaces, and modeling neurological disorders. However, realizing this vision requires scientific advances in stem cell bioengineering, neural interfaces, machine learning, and big data analytics, as well as anticipating ethical challenges around possible organoid consciousness and rights of cell donors. An interdisciplinary, multi-stakeholder collaboration is needed to confront the technical obstacles and ethical issues. The declaration urges the strategic development of OI as a field to unlock its benefits, but cautions that as organoids become more sophisticated, we must safeguard against emergent consciousness and protect donor interests through ongoing ethical discussions. In summary, the Baltimore Declaration calls for exploring organoid intelligence to propel neuroscience and biotechnology forward, while embedding ethics at each step of the way.

In conclusion, OI represents a radical approach to artificial intelligence that leverages advances in stem cell biology and bioengineering to implement intelligence based on actual human neural networks. Initial proof-of-concept studies demonstrate prospects for interfacing organoids with electronics to exhibit simple learning behaviors such as Kagan et al. (2022) described above or largely unpublished work by the Muotri group (Muotri, 2019; Pereira et al., 2023): Muotri’s team connected a brain organoid to a spider-shaped robot, allowing the organoid’s electrical activity to control the robot’s movements. The robot’s sensors detected when it was close to a wall, and the computer relayed those signals back to the organoid in the form of electrical pulses. In another experiment, Muotri’s lab taught a robot to walk and navigate its environment using the electrical activity from the neural oscillations of brain organoids. Substantial progress across many research fronts will be required to achieve the long-term vision of building advanced OI systems with meaningful real-world functionality. Through the pursuit of interdisciplinary and collaborative research guided by bioethical principles, the OI field aims to uncover new foundations for understanding biological intelligence and creating organically based computing.

## Brain organoids and organoid intelligence as the new ethical frontier

The use of brain organoids in research by itself raises ethical concerns.^1^ Several core ethical issues recur prominently in cerebral organoid research (Lavazza and Massimini, 2018; National Academies of Sciences, Engineering, and Medicine, 2021):

- Moral status and potential for consciousness or cognition
- Research oversight procedures and guidelines
- Informed consent standards for cell donors
- Human-animal neural chimeras
- Implantation into animal models
- Commercialization and intellectual property
- Public benefit and equitable access

Many of these apply to other fields of stem cell and organoid use in general, but the aspect of possible consciousness sharpens the discussion for brain organoids. Some experts argue that brain organoids can never develop consciousness, while others believe that higher moral status should never be attributed to them (Lavazza and Massimini, 2018; Sawai et al., 2022). We need discussions how the possibility that organoids develop consciousness or feelings of pain and suffering could be anticipated or precluded. At what level of complexity do such issues arise? What are the moral implications regarding the source of cells? How could donor consent processes adequately cover contributions to such novel intelligent systems? Who would have ownership over OI creations that integrate contributions across institutions and incorporate proprietary technology platforms? How can the disruptive effects of OI across society be prudently managed (Quirion, 2023), avoiding inequities in access to enhancements enabled by such technologies?

Clinical translation also raises added ethical questions around efficacy, safety, ownership, privacy, and equitable access that warrant deliberation. For example, expectations must be calibrated realistically regarding predictive validity compared to traditional preclinical models. Pressures to accelerate progress could overlook uncertainties in translating *in vitro* findings to humans. Furthermore, patient-derived organoids used for commercial drug development create tensions between public benefit and private profits (Kinderlerer, 2023) that could limit equitable availability of resulting therapies. More analysis is needed regarding how to responsibly balance scientific opportunities of OI-based drug screening with ethical imperatives to protect patient donors and ensure fair access. As applications expand, governance frameworks should proactively address these multifaceted translational issues.

A central question is the ethical implications of categorizing organoids as either subjects or objects (Munsie et al., 2017; Boers et al., 2019a,b). They argue that this binary categorization is problematic because it oversimplifies the complex moral value of organoids. Organoids have both ‘subject-like’ and ‘object-like’ values, and their moral value is more pluralistic than a simple divide between subject or object and gift or commodity (Boers et al., 2019a,b). This dual nature implies that organoids, as living models derived from human cells, possess characteristics akin to subjects (living entities with potential sentience or consciousness) and objects (inanimate things used for a specific purpose). Their moral value, therefore, is more nuanced and cannot be simply categorized as either a subject or an object, a gift or a commodity. This pluralistic view acknowledges the complex ethical landscape surrounding organoids, recognizing that they are not merely biological tools or commodities, but also potentially embody aspects of living, sentient beings. The paper also addresses the ethical issues related to the commercialization of organoids. The authors argue that the commercialization of organoids is legitimized by a detachment of the instrumental and commercial value of organoids from their associations with persons and their bodies. This detachment is enacted in steps of disentanglement, among which consent, and commodification play a significant role. The authors contend that far-reaching disentanglement is ethically challenging because societal interests could be put under pressure, and the interests of donors are made subordinate to those of third parties. They propose a ‘consent for governance’ model that contributes to responsible innovation and clinical translation in this field. In response, Lavazza (2019) stressed that with increase in complexity to “minibrains,” we must thoughtfully consider if and when they develop sentience and how to ethically limit harms. Furthermore, obtaining meaningful informed consent from donors must ensure they retain some long-term control over their genetic material used to create organoids. Finally, we must ensure organoids derived from genetic minorities are used equitably and not in ways that perpetuate discrimination or stigma.

Ethical issues surrounding brain organoid research include formal oversight, procurement of human biomaterials, translational delivery, animal research, and consciousness/moral status (Hyun et al., 2020). As organoids advance toward greater complexity, ethical concerns may arise about potential consciousness, though current evidence suggests this is unlikely (Farahany et al., 2018; Lavazza and Massimini, 2018). Concerns also exist around human-animal chimeras if organoids are engrafted in animals, based on effects on animal welfare and moral status (Munsie et al., 2017; Mansour et al., 2018). Robust informed consent is needed for biomaterials and data sharing as organoids are increasingly used for personalized medicine (Boers and Bredenoord, 2018; Boers et al., 2019a,b). Commercialization and equitable access are additional ethical considerations (Boers et al., 2019a,b). Open questions remain regarding if standard oversight mechanisms sufficiently cover brain organoids versus need for adapted guidelines and review processes (Cheshire, 2014). Overall, continued dialog among scientists, bioethicists, regulators, and publics can foster responsible innovation in this promising field.

Lavazza (2021a,b) sees the ethical arguments related to brain organoid falling into the main themes of research oversight, consciousness, interaction with social environment, formal ethical oversight, and implantation in animals. This needs to be weighed against animal welfare and rights, and concerns related to the use of nonhuman primates they replace. A framework known as the Six Principles (6Ps) (Beauchamp and DeGrazia, 2020; DeGrazia and Beauchamp, 2021) aims to expand upon the 3Rs and be a practical means for assessing animal ethical issues like neural-chimeras and cerebral organoid xenotransplantation (Barnhart and Dierickx, 2023). In her 2021 thesis “*Protecting In Vitro Human Brain Organoids: Why, When, and Which?*,” Das (2021) delves into the ethical implications of using human brain organoids in research. She discusses the complex nature of their moral status, suggesting it hinges on factors such as structural complexity and potential cognitive abilities, including consciousness and the ability to experience emotions. Das debates the adequacy of sentience as a sole determinant of moral status. She also explores various ethical models for conducting research with brain organoids, such as Kantian theory and applying animal research ethics, while acknowledging the limited likelihood of significant consciousness in these organoids.

*The Emerging Field of Human Neural Organoids, Transplants, and Chimeras: Science, Ethics, and Governance* is a report by the National Academies of Sciences, Engineering, and Medicine (2021) that reviews the status of research, considers its benefits and risks, discusses associated ethical issues, and considers governance mechanisms for this type of research. The report identifies three models of human brain research: human neural organoids, human neural transplants, and human-animal neural chimeras. The use of brain organoids in research raises ethical concerns; these include the possible need for stringent research restrictions and formal ethical oversight for advanced brain organoids as well as aspects of consciousness, interaction with social environments, and implantation in animals (de Jongh et al., 2022; Hoppe et al., 2023). Researchers, policymakers, and bioethicists are called upon to work together from the early stages of research and development onwards to identify emerging ethical questions and take new directions. A systematic review of ethical issues associated with organoids identified brain organoids, chimeras, and transplantation of human-derived organoids into animals as specific sub-types of organoids that raise ethical concerns. The National Academy report (2021) also points out the need to define the distinctions between human beings and other animals, animal welfare and rights, and the potential for consciousness and enhanced capacities in research animals or neural organoids. The International Society for Stem Cell Research (ISSCR) recently released guidelines minimizing ethical concerns about human-animal chimera research (Koplin, 2022), contradicting other major reports warning that chimeras with humanized brains could attain enhanced moral status. The ISSCR justifies its permissive stance using controversial philosophical assumptions that could also dismiss moral constraints on practices like infanticide. This suggests the ISSCR guidelines rely on unstable foundations and fail to address core issues around conferring moral status through cognitive enhancement.

The National Academy report (2021) discusses the ethical implications of using cells derived from biological materials of persons who did not know their materials were being used for such research and would have objected if they were. Ethical collection of human biomaterials for organoid derivation requires rethinking informed consent, privacy protection, and data sharing approaches (Boers and Bredenoord, 2018). With increasing commercialization, intellectual property issues and equitable access to benefits must be addressed (Roberts et al., 2014).

Sawai et al. (2022) provides a comprehensive discussion on the ethical implications of brain organoid research. The authors argue that the moral status of brain organoids becomes a concern when these organoids exhibit features associated with consciousness. They propose a framework for assessing these ethical issues, which includes the potential for consciousness, the moral status of organoids, and the application of the precautionary principle. The precautionary principle suggests that if an action or policy has the potential to cause harm to the public or the environment, in the absence of scientific consensus, the burden of proof falls on those advocating for the action or policy. Furthermore, the authors suggest that the development of guidelines and regulations is necessary to address these ethical issues. They also highlight the need for ongoing dialog among scientists, ethicists, policymakers, and the public to navigate the ethical landscape of brain organoid research (Sawai et al., 2022).

Lavazza and Reichlin (2023) discuss the ethical implications of research grafting or transplanting into rodent brains, to ensure vascularization and a more suitable growth environment. This showed that organoids transplanted into the brain of a host not only lived a long time and integrated into the brain environment without harming the animal but also integrated structurally and functionally into it (Mansour et al., 2018; Chen et al., 2019; Revah et al., 2022; Vermaercke et al., 2022). There are obvious moral concerns of using human brain matter in devices, particularly when the user is aware that they are interacting with a device forming a human “mini-brain” grown from the cells of a donor (Inglese and Lavazza, 2022). Lavazza and Reichlin (2023) argue that there is no moral argument *per se* against using lab-grown human cells to improve the performance of an artifact. However, the moral sensitivity of most users, who would have moral concerns about the commercial and purely instrumental use of complex brain organoids, is a significant factor (Kateb, 2014). As organoids increase in sophistication, ethical issues arise regarding organoid-animal chimeras and potential organoid consciousness (Lavazza and Massimini, 2018; Shepherd, 2018; Hoppe et al., 2023). When organoids are transplanted into animal hosts, human-animal chimeras can be created. Monitoring for enhancements like cognition or self-awareness in chimeras is important, as is considering the moral status of enhanced animals (Chen et al., 2019; Hoppe et al., 2023). Stringent oversight is needed for chimera studies. If organoids develop primitive forms of consciousness, this raises questions about their moral status and whether they could experience pain or suffering (Lavazza, 2021a,b). Assessing consciousness through methods like integrated information theory and tests for neural correlates of consciousness may be necessary (Tononi et al., 2016).

Many of these discussions are aggravated when research is aiming at realizing intelligence in brain models. The foundational paper “*Organoid intelligence (OI): the new frontier in biocomputing and intelligence-in-a-dish*” by Smirnova et al. (2023a) discusses several ethical considerations related to the use of organoid intelligence in research:

1. *Public perception and engagement*: The authors emphasize the importance of understanding public perceptions of organoid intelligence and argue that this understanding cannot be delegated to ethicists alone. They propose a three-way feedback loop involving researchers, ethicists, and members of the public, including stakeholders who could be especially impacted by advances in organoid intelligence. This feedback loop would enable specific applications of organoid intelligence to be articulated by researchers, analyzed by ethicists based on theoretical principles, and evaluated by members of the public with diverse moral perspectives.
2. *Privacy and intellectual property*: The authors also highlight potential privacy concerns on the part of induced pluripotent stem cell (iPSC) donors and aspects of intellectual property. They pose questions such as what the organoid might reveal about the cell donor, whether there is a moral obligation to inform the donor if something relevant to their health is identified during research, and whether donors have rights that extend beyond the donation.
3. *Embedded ethics*: The authors propose to use an “embedded ethics” approach whereby an ethics team will identify, discuss, and analyze ethical issues as they arise in the course of this work. Embedded ethics is a standard approach in interdisciplinary ethics research, whereby expert ethicists join and collaborate integrally with research and development teams to consider and address ethical issues via an iterative and continuous process as the research evolves.

The Frontiers Policy Outlook, a feature of Frontiers Policy Labs, i.e., opinion pieces written by key policy experts, providing a policy perspective on a specific lead article published in Frontiers in Science, titled “*Organoid Intelligence: Society Must Engage in the Ethics*” by Kinderlerer (2023) emphasizes the importance of engaging in ethical discussions and involving diverse stakeholders to ensure responsible development and use of organoid intelligence. The ethical and social responsibility requires the suggested “embedded ethics” approach to ensure that OI develops in an ethically and socially responsible manner. This approach involves interdisciplinary teams of ethicists, researchers, and members of the public working together to identify, discuss, and analyze ethical issues. Kinderlerer explores the ethical and legal issues related to brain organoids that may develop cognitive properties. This includes considerations of human dignity and the rights of both donors and organoids. An interdisciplinary collaboration is crucial to address the ethical concerns associated with OI. The proposal for creating “intelligence in a dish” using human brain cells to perform advanced tasks raises ethical concerns and highlights the need for careful consideration of the implications and potential risks associated with such technology.

This article aims to expand these discussions in view of the increasing traction of the OI concept, which for example was instantly included into the *BOLD GOALS FOR U.S. BIOTECHNOLOGY AND BIOMANUFACTURING*^2^ and shall become a National Science Foundation “*Engineering Organoid Intelligence*” program.^3^

## Conceptualizing consciousness and the mind-brain relationship

Brain organoids intersect longstanding philosophical debates on mind, consciousness, and moral status. A key question is whether organoids could possess intrinsic moral worth based on their neurobiological properties, rather than merely instrumental value as research tools. Some argue that only beings with subjective experiential states warrant moral status, whereas others propose cognitive capacities like reasoning confer status (Lavazza and Massimini, 2018). Lavazza and Massimini (2018) propose a method to measure consciousness in brain organoids. Cerebral organoids clearly lack capacities for consciousness or cognition currently. Some authors claim that the morphology and function of organoids are sufficient to exclude the presence of consciousness (Lunshof, 2020). However, their anticipated future abilities raise concerns about possible moral considerability emerging during research, necessitating ethical oversight (Owen et al., 2023).

Of course, possession of human neurons alone does not confer moral status. The developmental context matters (Farahany et al., 2018). In contrast to natural embryological development, cerebral organoids are artificially engineered from stem cells. This teleological creation for human purposes bears on assessing their moral standing. Furthermore, realizing the promise of cerebral organoids to benefit human health provides a strong utilitarian rationale. Ethical frameworks must weigh all factors, including moral duties to patients that may justify purposeful creation of limited cerebral organoid capabilities.

The unprecedented ability of cerebral organoids to model aspects of the developing human brain also necessitates conceptual clarification on notions of consciousness, mind, and personhood.

### Defining consciousness

Consciousness remains enigmatic, lacking consensus on necessary or sufficient criteria (Bayne, 2010; Bayne et al., 2016; Koch et al., 2016). The term carries multiple meanings, ranging from basic awareness of stimuli to higher-order self-consciousness. Most definitions require some form of sentience, phenomenal experiences, and integration of information. But theories diverge on the specific mechanisms, with proposals highlighting recurrent processing, global workspace integration, higher-order representations, or causal density like integrated information theory (Bayne et al., 2016; Tononi et al., 2016). Empirically assessing consciousness also presents challenges. Behavioral responsiveness is used for clinical evaluation, while neuroimaging methods allow inferences about internal awareness in unresponsive patients (Boly et al., 2008; Naccache, 2018). However, these approaches cannot directly measure subjective phenomenal experience. Applying such measures meaningfully to assess consciousness in organoids will require comparative validation.

### Mind-brain relationship

Understanding mind-brain relationships is intertwined with mapping attributes of consciousness to properties of neural systems (Kandel et al., 2013). However, the precise linkage between mental states and brain activity remains elusive. Neuroscience depicts the mind as emerging from the collective interactions of billions of neurons that instantiate cognition and consciousness. However, the precise linkage between mental states and brain activity remains little understood (Chalmers, 1996; Tononi and Koch, 2015). Most agree that subjective experience and consciousness require certain structural and functional complexity in a neural system. But theories diverge on whether sensory activity, recurrent processing, widespread integration, certain oscillatory patterns, or specific cellular mechanisms are most crucial. The development of cerebral organoids could provide platforms to investigate mind-brain relationships and assess which factors may give rise to consciousness. By systematically manipulating and monitoring organoid complexity, sensory inputs, information integration, and neural patterning, researchers may gain insights into which factors are necessary or sufficient for conscious awareness to arise. This could shed light on theoretical debates about whether morphological, functional, or informational properties of neural systems give rise to consciousness. However, organoids are currently certainly too rudimentary to recreate full human consciousness (Goldfine and Schiff, 2011). Their future potential to cross this threshold, as research advances, raises important ethical and legal questions.

### What is intelligence?

**Figure 1:**
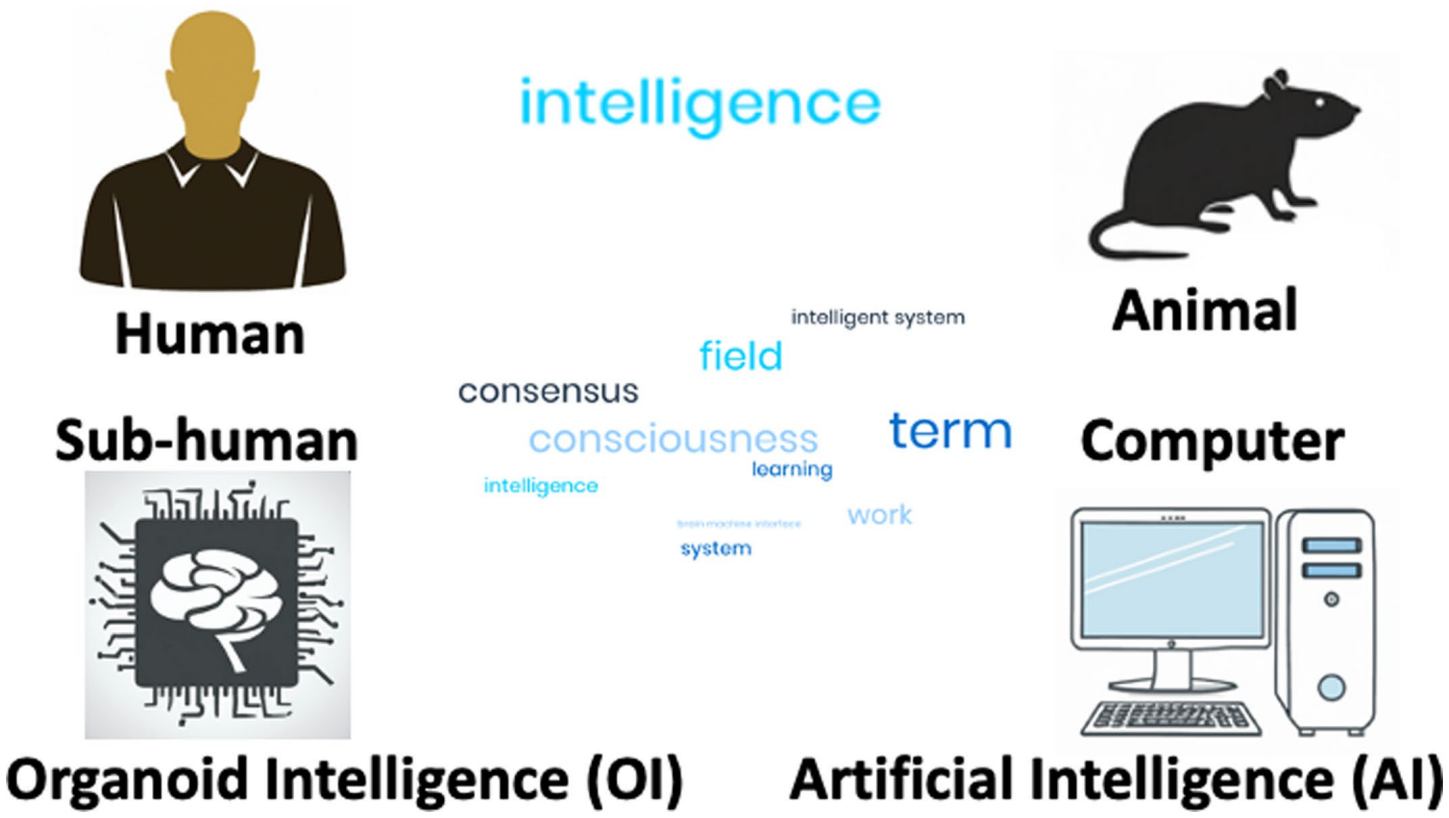
Various incorporations of intelligence. Intelligence is shown as a world field to indicate that the term is difficult to define by consensus and means very different things in the context of the four embodiments discussed.

Arguably, intelligence is what makes us human. We do not want to enter discussions that unintelligent humans still have human rights, and that the absence of intelligence does not mean that animals should not be protected. More than memory and learning or consciousness, intelligence is a critical distinction between humans, non-human animals and the emerging more or less intelligent embodiments of computational Artificial Intelligence (AI) and OI (Figure 1).

The rise of AI has started a discussion about when to consider these systems intelligent. Laskar (2023) argues that genuine machine intelligence does not simply rely on traditional machine learning or neural networks but on causal learning and inference algorithms. To develop truly intelligent machines, it is imperative to teach them how to interact with the world, moving beyond pattern recognition to understanding causal relationships. True machine intelligence is represented by causal models of the world, not mere statistical models.

Deep learning has achieved remarkable results in recent years, attaining state-of-the-art performance in tasks like image recognition, speech processing, and natural language processing. However, these powerful deep neural networks suffer from a lack of transparency – they operate as “black boxes” that do not provide explanations for their predictions (Ridley, 2019). This presents challenges for trust, verification, and transferability to new situations. Genuine causality is at the heart of common-sense reasoning and scientific knowledge. To increase transparency, various methods have been proposed to make deep learning more interpretable (Samek et al., 2017). Techniques like saliency maps highlight parts of the input most relevant to a model’s output. Approaches like LIME (*Local Interpretable Model-agnostic Explanations*, is a technique that approximates any black box machine learning model with a local, interpretable model to explain each individual prediction) approximate a complex model locally with a simple, interpretable model to explain individual predictions. Methods like SHAP (*Shapley Additive exPlanations*, a game theoretic approach to explain the output of any machine learning model) assign each input feature an importance value for a particular prediction. However, while these methods increase model transparency retrospectively, inherently interpretable models offer greater explainability by design. Decision trees and rule-based systems encode clear logical relationships in their structure. Attention-based architectures like transformers explicitly model variable dependencies. Capsule networks represent hierarchical relationships between parts and wholes. Future progress in deep learning transparency will likely involve a combination of post-hoc explanation methods and inherently interpretable architectures. The development of causally-driven neural networks, which represent causal variables and relationships, is a promising direction (Pearl, 2019; Schölkopf, 2019). Integrating graphical causal models with deep learning can produce AI systems that offer robust explanations aligned with human reasoning. Improved transparency will accelerate the development and adoption of trustworthy and ethical AI.

Understanding causal relationships is critical for assessing and demonstrating intelligence and cognition in organoid systems. On the AI side, causal inference capabilities are hallmarks of advanced machine learning systems that can reason about the world and make reliable predictions. Models based purely on pattern recognition have limitations in generalizability and interpretability. Integrating causal graphs and models allows AI systems to better capture the complex dynamics of real-world phenomena. Likewise, attributing intelligence to organoids requires moving beyond simple input–output mappings to analyze the underlying causal mechanisms. Organoids exhibit spontaneous neural activity, but true cognition likely requires directed information processing and decision making grounded in causal mental models.

Mapping different types of causal connections in organoid networks could provide insights into their computational capabilities. For instance, demonstrating common-cause relationships where a stimulus triggers downstream effects would show integration of sensory signals. Causal chains that transform inputs into behavior via intermediate representations would illustrate richer information processing. Cyclical causal patterns may emerge as organoids develop recurrent, self-reinforcing circuits underlying memory and learning. By relating organoid connectivity and dynamics to these markers of causal cognition, we can better evaluate claims of intelligence. Combined with AI to analyze and interact with organoids, causal mapping provides a rigorous framework for testing and enhancing biological cognition. Integrating causal inference in both software and wetware components is key to realizing the vision of organoid intelligence. In the context of OI, this must be applied both to the AI-based data analysis and in translation to the proof of intelligent behavior of the organoid. There are different types of Causal Representation:

- Linear direct causality: This refers to a straightforward cause-effect relationship.
- Common-cause relationships: One cause leading to multiple effects.
- Common-effect relationships: Multiple causes leading to a single effect.
- Causal chains: One cause leads to an effect which then becomes a cause for another effect.
- Reverse causality: In complex systems or situations where the distinction between causes and effects is not straightforward, leading to a cycle where causes and effects interchangeably influence each other.
- Causal cycles: A concept common in systems thinking, where a series of interconnected events or conditions so that the outcome of one event influences another, and this in turn affects the initial event, creating a loop of cause and effect. In these cycles, events are both causes and effects within the same system, often reinforcing or amplifying each other.
- Full causation: This encompasses all possible causal relationships and is represented by full causal undirected cyclic graph networks, which can incorporate known and unknown neural network architectures.

In conclusion, for AI and in consequence OI to mirror human intelligence and understanding, it must grasp and interpret causality, not just recognize patterns. This shift toward causality, on the one hand, will make AI more adaptable, transparent, and effective in real-world applications, and on the other hand will prove and make exploitable intelligent OI behavior.

## Ethical and legal issues

The very factors limiting fidelity to the human brain also shape the ethical debate on cerebral organoids. A key question is whether they could attain capabilities resembling human consciousness, emotions, or cognition. This hinges on unresolved theories of mind and consciousness. While neuroscience has extensively mapped correlates of consciousness, there is no consensus on how consciousness arises in the brain (Koch et al., 2016). Integrated information theory, global neuronal complexity, and global workspace theory offer different perspectives. Most theories emphasize whole-brain integration, which embryonic cerebral organoids lack. Nonetheless, assessing the potential for consciousness remains challenging without a definitive understanding.

Experimental practices also bear on ethical considerations. Cerebral organoids are specifically engineered from stem cells, rather than developing naturally. This teleological context differs morally from human embryos. Purposefully enhancing organoid complexity requires weighing scientific benefits against ethical risks (Farahany et al., 2018). For instance, xenotransplantation into rodents raises concerns about human neural chimeras (Yang et al., 2020). While the extent of functionality and sentience remains unsettled, ethical caution may be warranted as engineering aims closer toward recapitulating advanced human neural capabilities.

Cerebral organoids can elicit moral concern based on their human neural cell identity and resemblance to developing brains, unlike other stem cell-derived organoids (Farahany et al., 2018). Ethical issues arise regarding organoids’ moral status, human-animal chimeras, informed consent, and comparisons with animal research.

### Moral status

Moral status denotes whether an entity warrants ethical consideration based on certain attributes like sentience (Basl and Honnefelder, 2020). The moral status of brain organoids is complex, depending on properties like physical substrate, cognitive/conscious capacity, and sensory ability (Lavazza and Massimini, 2018). While current organoids likely lack moral status akin to human persons, their status could change as research advances if they develop sophisticated neural structures and causal interconnectivity that approaches conscious awareness (Farahany et al., 2018; Lavazza and Massimini, 2018). However, purely structural criteria like counting neurons are insufficient to confer moral status, which instead depends on functional capacities like sentience (Lavazza and Massimini, 2018). The moral status debate warrants continued reassessment as organoid properties evolve.

### Human-animal chimeras

Transplanting human brain organoids into animal models raises concerns if it significantly alters the recipient’s cognitive functions or identity (Mertes et al., 2017; Singh et al., 2019). However, current organoids lack capacity for high-order functioning, and most transplantation studies report integration without widespread colonization or functional alteration (Sloan et al., 2017; Mansour et al., 2018). Though organoids might engraft locally, strong scientific justification is still needed before allowing advanced humanized chimeras. Oversight is required to evaluate proposed human-animal chimera experiments (Farahany et al., 2018; Hyun et al., 2021).

### Informed consent

Obtaining meaningful informed consent from donors is critical when collecting human biomaterials to generate organoids (Boers et al., 2019a,b; Hyun et al., 2020). Consent documents should clearly explain foreseeable uses of donor cells for organoid research, including development into complex assembloids or human-animal chimeras (Andersen et al., 2020). Donors should have the ongoing option to withdraw consent as new applications arise (Boers and Bredenoord, 2018; de Jongh et al., 2022). Continuous engagement through participatory deliberation models can align organoid advancement with societal values (Bredenoord et al., 2018). Several issues warrant consideration regarding consent for organoid derivation: (1) Scope and duration of permission for use of biomaterials, (2) Options and processes for donors to withdraw or restrict consent (Boers and Bredenoord, 2018), (3) Ownership, commercialization, and intellectual property issues (Boers et al., 2019a,b) and (4) Privacy risks from genomic or medical data generated. Communication of uncertainties and ability to re-consent for unforeseen uses. Transparency about such issues can improve trust in biobanking and help ensure consensual stewardship (Lewis and Holm, 2022). Accessible engagement and participatory oversight should supplement one-time consent to promote responsible innovation (de Jongh et al., 2022). Implementing Dynamic Consent platforms could also enable donors to manage permissions amid evolving organoid applications (Kaye et al., 2018).

### Animal research ethics

Parallels exist between organoid research ethics and animal research ethics; similar ethical principles of reducing harm, optimizing welfare, and non-maleficence apply (Greene et al., 2005; Farahany et al., 2018). Concepts like the 3Rs framework are relevant for setting organoid-specific guidelines minimizing unnecessary procedures or discomfort (Tesar et al., 2021). However, unique issues like moral status also require tailored governance.

Brain Organoids must be seen also in Translational Contexts of their medical use: Cerebral organoids could for example transform personalized medicine for neuropsychiatric disorders by enabling patient-specific disease modeling and drug screening. The prospect of clinical translation, however, brings added ethical dimensions regarding efficacy and safety testing compared to basic research uses (Sloan et al., 2018). Psychiatric treatment often involves risky and poorly understood medications. Preclinical testing in non-human models has frequently failed to predict pharmacological effects and side-effects in humans (Pistollato et al., 2016; Van Norman, 2019). Brain organoids offer hope as more faithful testbeds before human trials, but validating predictive value requires further comparator studies. Pressures to accelerate clinical translation should be carefully weighed against uncertainties.

Patient-derived organoids also introduce questions of ownership, privacy, and consent (Boers et al., 2019a,b). Biobanks of patient-derived cerebral organoids, for instance, represent sensitive personal biological data. Consent processes should transparently address potential dual uses for research and clinical care. Commercial entities are also entering the cerebral organoid field, attracted by prospects of personalized therapies. This raises additional concerns around commodification and equitable access that merit attention (de Jongh et al., 2022).

The novel applications and ethical issues associated with human brain organoids necessitate an assessment and potential expansion of legal frameworks. Several concerns such as moral status, consent standards, and data privacy are raised, but additional legal considerations warrant deliberation. For instance, the current legal status of organoids and any rights owing to them remain unclear, but this may need to be addressed if evidence of sentience emerges. Furthermore, the global landscape varies regarding laws governing embryo research, human-animal chimera studies, and other ethically complex areas that intersect with organoids. Thus, harmonizing standards across different jurisdictions will pose challenges but promote responsible innovation. Updates to property laws, data protection statutes, and specialized regulations may be prudent to align with rapidly advancing organoid capabilities. Legal experts should be proactively engaged alongside scientists and ethicists to evaluate where existing laws are insufficient. The potential for unprecedented applications warrants an evolving governance approach responsive to ongoing legal gaps. A flexible but principled legal foundation for organoid research can help realize benefits ethically.

## Governance and regulation

The ethical discussions summarized so far might need to be translated into regulation and governance. A special status beyond other types of organoids would result from possibly achieving consciousness. Therefore, there is a need to discuss possibly necessary research restrictions for advanced brain organoids than for organoids that cannot plausibly possess consciousness (Sawai et al., 2022). Most current models without perfusion like our own (Pamies et al., 2017) are restricted to sizes of below half a mm, which means the comprise less than 100,000 neurons compared to close to 86 billion estimated for a human brain (Azevedo et al., 2009). Brain organoids cannot interact with a human social environment in the laboratory, which raises questions about their ability to develop consciousness (Lavazza and Massimini, 2018; Reardon, 2020). The study of organoids, however, falls into an odd gap between other areas of research, complicating formal ethical oversight (Reardon, 2020). There are also ethical concerns surrounding the implantation of human brain organoids into animals, which could raise questions about their intelligence, level of consciousness, and species identity (Hyun et al., 2016; Sawai et al., 2022). Scientists, policymakers, and bioethicists are called upon to work together from the early stages of research and development onwards to identify emerging ethical questions and take new directions (Sawai et al., 2022). A comprehensive ethical framework is needed to guide research in this area, balancing interests, values, and principles (Zilio and Lavazza, 2023). Hoppe et al. (2023) emphasize that organoid advances require parallel ethical deliberation among scientists, regulators, and the public regarding the acceptability of brain organoid research directions. A thoughtful code of conduct is needed to ensure ethical scientific progress (Owen et al., 2023; Zilio and Lavazza, 2023).

The central concern is determining what kinds of oversight are owed to advanced brain organoids. This is interlinked with assessing capacities for consciousness or cognition. Current evidence suggests only simple neural activity and connectivity possible, but the situation could change as engineering aims deliberately toward more sophisticated capabilities. Regular ethics review and specialized oversight guidelines have been widely proposed for such scenarios (Farahany et al., 2018). Several authors have called for special ethical guidelines or regulatory frameworks for clinical research using brain organoids to ensure the welfare of brain organoids in the future (de Jongh et al., 2022). The need for stringent research restrictions for advanced brain organoids, versus concerns about animal welfare and rights, and concerns related to the use of non-human primates (Hyun et al., 2020; Reardon, 2020).

Informed consent standards constitute another key issue (Boers et al., 2019a,b). Donors contributing biological materials for cerebral organoids should understand how these could be used, including development into complex multi-regional assembloids (Andersen et al., 2020) or human-animal chimeras. The possibility of opting out merits consideration. Transparency in consent processes will help maintain public trust. The novel capabilities and ethical issues associated with human brain organoids necessitate adapted governance to align with public values while enabling research (Farahany et al., 2018). This could include self-regulation by researchers, adapted research ethics oversight, guidelines tailored to organoids, and clarity on legal dimensions.

### Adapting oversight

Standard research ethics committees provide foundational review but lack specialized expertise to evaluate all nuances of novel cerebral organoid experiments (Munsie et al., 2017; Farahany et al., 2018). Adapted governance might incorporate input from neuroethicists and philosophers to identify potential issues, applying concepts like the precautionary principle for higher-risk protocols (Boers and Bredenoord, 2018). Extension of oversight models for human embryo research could be considered for organoid studies bordering on moral concern thresholds (Munsie et al., 2017; Hyun et al., 2021).

### Legal dimensions

The legal status of brain organoids and any rights owing to them remain unclear currently but warrant deliberation as research progresses (Farahany et al., 2018; Lavazza and Massimini, 2018). Property rights and usage restrictions on donated biomaterials used to generate organoids also require clarification (Boers et al., 2019a,b). Data protection and privacy are additional legal considerations, given the personal nature of organoids derived from individuals’ cells.

### Guideline development

Formulating adapted guidelines can outline ethical requirements, boundaries, and best practices tailored to cerebral organoid research across various translational phases (Kimmelman et al., 2016; Munsie et al., 2017). This can provide researchers a decision-making framework regarding permissible applications. Such guidelines will likely require iterative refinement as both the science and ethics evolve.

### Governance

The regulatory landscape for cerebral organoids varies internationally, shaped by different scientific, cultural, and ethical perspectives. In the United States, NIH guidelines restrict federal funding of human-animal chimeric research, but few formal policies otherwise exist. The UK similarly lacks specific regulation, instead relying on general governance frameworks for medical research and human tissues (National Academies of Sciences, Engineering, and Medicine, 2021). Germany’s ethics traditions impose more constraints on research deemed contrary to human dignity, which could impact organoid manipulation (Pichl et al., 2023). No international consensus has emerged yet on cerebral organoid governance.

Establishing international consensus on organoid oversight should become a priority. Global harmonization can enable responsible innovation and application of consistent ethical standards across diverse settings. Organizations well-positioned to spearhead these dialogs include the World Health Organization (WHO), which could issue guidance through its Ethics Review Committee (ERC), and the United Nations Intergovernmental Bioethics Committee. Initial priorities for concord include governing areas like consent protocols, embryo/chimera research review processes, restrictions on complex cognitive organoids, and data sharing norms. By first targeting issues with clear ethical urgency, broad norms can crystallize to guide emerging research directions. Within 5–10 years, additional governance matters warranting consideration include commercialization policies balancing access and intellectual property protections, guidelines for advanced neural interfacing studies, and frameworks for equitable benefit sharing globally. Preemptive international collaboration across science ministries can uphold ethical priorities despite differing cultural perspectives. If addressed proactively, even complex questions of organoid legal status, rights, and moral standing can become more tractable through multilateral cooperation. Overall, the rapid pace of organoid advancement underscores the need for swift action toward ethical consistency worldwide.

## Public perceptions and science communication

The first level of communication is to the patients, who must donate cells only with informed consent. Bollinger et al. (2021) discuss several ethical issues regarding organoid research from the patient perspective:

1. Therapeutic misconception risks from misunderstanding experimental nature
2. Need for explicit consent encompassing diverse uses of donated biomaterials
3. Calls for appropriate oversight and adherence to ethics guidelines
4. Concerns about profit motivations and equitable access with commercialization

The authors emphasize thorough informed consent processes to avoid misperceptions, formal governance to ensure ethical standards, and concerns that industry profit motives could limit patient benefit. They suggest patients are more comfortable with academic or government institutions leading this research aimed at helping people.

On a second level, there is the communication to a general public. Public perceptions also differ across countries. Religion and taboos regarding human-animal mixing may evoke visceral reactions in some populations (Lavazza and Massimini, 2018). Metaphors equating organoids with “mini-brains” could fuel unrealistic expectations or fears. Responsible governance should incorporate public values and avoid misleading hype. Overall, transparent cross-cultural dialog on cerebral organoid research will be vital for developing ethically grounded, universally acceptable policies. The National Academies report (National Academies of Sciences, Engineering, and Medicine, 2021) suggests that these issues require discussion among people with different perspectives. They also highlight the importance of engaging with the public, understanding and addressing their concerns, and linking research to important unmet needs, such as the need for treatments for brain diseases. Existing surveys reveal general support among publics for brain organoid research aimed at reducing suffering from disease, accompanied by concerns about consciousness, chimeras, and commercialization (Haselager et al., 2020; Steinsbekk et al., 2021). However, most laypersons lack awareness about organoids, and perceptions are readily shaped by hype or unrealistic media portrayals (Haselager et al., 2020). Responsible science communication explaining the state of the field while acknowledging current limitations and uncertainty is needed. Scientist engagement with publics and ethicists can help align organoid technology trajectories with societal values and risk–benefit judgments (Bredenoord et al., 2018).

On a third level, there is the communication among scientists in order to self-regulate. This can be exemplified by the work of EuroStemCell,^4^ which is a partnership of more than 400 stem cell and regenerative medicine labs across Europe. Some of the key areas of ethical debate regarding brain organoids they see include (de Jongh et al., 2022):

- *Research oversight*: There is a need for stringent research restrictions for advanced brain organoids than for organoids that cannot plausibly possess consciousness.
- *Consciousness*: Some experts argue that brain organoids can never develop consciousness, while others believe that higher moral status should never be attributed to them.
- *Interaction with social environment*: Brain organoids cannot interact with a human social environment in the laboratory, which raises questions about their ability to develop consciousness.
- *Formal ethical oversight*: The study of organoids falls into an odd gap between other areas of research, complicating formal ethical oversight.
- *Implantation in animals*: There are ethical concerns surrounding the implantation of human brain organoids into animals, which could raise questions about their intelligence, level of consciousness, and species identity.

EuroStemCell call on scientists, policymakers, and bioethicists to work together from the early stages of research and development onwards to identify emerging ethical questions and take new directions.

## Conclusion

Cerebral organoids offer unprecedented access to early human neurodevelopment, with tremendous potential to elucidate psychiatric disorders and realize personalized therapies. However, possibilities of engineering sophisticated capabilities require proactive ethical foresight. We have outlined key scientific, philosophical, and ethical dimensions needing consideration. A nuanced governance framework should be developed through open international deliberation, weighing all perspectives.

Two recent publications were brought to the authors’ attention while finalizing this manuscript. Kagan et al. (2023) describe recent work developing “*Synthetic Biological Intelligence*” (SBI) systems that integrate biological neural networks grown *in vitro* with digital computing. They highlight how modern stem cell technology enables scaling up neuronal cultures, advances in hardware/software allow real-time interaction with these cultures, and computational theories guide eliciting intelligent behaviors. As a proof-of-concept, they show embodied cortical neurons learning to play a video game through closed-loop electrical stimulation. Kagan et al. emphasize the need for an ethical framework to ensure responsible development of SBI technology. They propose addressing issues around terminology standardization, identifying metrics to track morally relevant properties like consciousness, and adopting an “*anticipatory governance*” approach that engages diverse stakeholders to steer applications toward desirable outcomes. Their work aligns with the emerging field of OI in seeking to leverage biological neural systems for enhanced computing.

Boyd and Lipshitz (2024) focus specifically on debates regarding consciousness and moral status of human brain organoids. They present a conceptual framework identifying four features grounding moral status: evaluative stance, self-directedness, agency and other-directedness. Consciousness matters morally if it enables these capacities. The paper maps comparative empirical approaches to study animal consciousness onto organoid research, arguing such behavioral studies are needed to attribute moral status to organoids. Until such investigations are conducted, they caution against simply assuming organoids have morally relevant consciousness based on neuroanatomical properties alone.

The perspectives of Kagan et al. and Boyd et al. reinforce key points from this paper. All three papers concur that realizing the promise of Organoid Intelligence requires parallel ethical deliberation among diverse stakeholders, emphasizing public engagement and transparent communication. They highlight consciousness as a pivotal consideration, but caution against simplistic assumptions that any neural complexity confers moral status. Careful empirical study of organoid functionality using behavioral measures adapted from animal cognition research will likely be needed. Overall, these works call for an anticipatory, adaptive governance approach balancing ethical caution and scientific progress through collaborative, interdisciplinary review.

Realizing the promise of human brain organoids for medicine requires navigating complex ethics tensions and societal perceptions. An interdisciplinary framework incorporating scientific, philosophical, ethical, legal, and public perspectives can steer organoid research toward responsible translation. Lavazza and Reichlin (2023) called for the extended scientific community, neuroethicists, and other relevant experts to contribute to the development of this ethical framework, in dialog with society and policymakers (Bassil and Horstkötter, 2023; Capps, 2023). Creative governance solutions and proactive engagement fostering mutual understanding between scientists and publics will be key. With prudent guidance, organoids could unlock profound insights into the brain while aligning with deeply held societal values. With thoughtful guidance, this promising field can progress rapidly within ethical bounds.

## Author contributions

TH: Conceptualization, Visualization, Writing – original draft, Writing – review & editing. IM: Writing – review & editing. LS: Writing – review & editing.

## Footnotes

^1^https://www.neuroethicssociety.org/oxford-meeting-2018

^2^https://www.whitehouse.gov/wp-content/uploads/2023/03/Bold-Goals-for-U.S.-Biotechnology-and-Biomanufacturing-Harnessing-Research-and-Development-To-Further-Societal-Goals-FINAL.pdf

^3^https://www.nsf.gov/news/news_summ.jsp?cntn_id=307280&org=EFMA

^4^https://www.eurostemcell.org/ethics-brain-organoids
